# World Health Report on Vision: Aging Implications for Global Vision and Eye Health

**DOI:** 10.1093/geroni/igaa057.2933

**Published:** 2020-12-16

**Authors:** Bonnielin Swenor, Varshini Varadaraj, Moon Jeong Lee, Heather Whitson, Pradeep Ramulu

**Affiliations:** 1 Johns Hopkins University, Baltimore, Maryland, United States; 2 The Wilmer Eye Institute, Johns Hopkins University School of Medicine; Department of Epidemiology, Johns Hopkins Bloomberg School of Public Health, Baltimore, Maryland, United States; 3 Duke University, Durham, North Carolina, United States; 4 Johns Hopkins School of Medicine, Baltimore, Maryland, United States

## Abstract

In 2019, the World Health Organization World Report on Vision estimated that that 2.2 billion people have a vision impairment, of which almost half could have been prevented or is yet to be addressed. As the global population ages and the prevalence of visual impairment increases, inequities in eye care and the downstream health and aging consequences of vision loss will become magnified. This session will: (1) provide key information regarding the burden of eye disease and visual impairment among older adults worldwide; (2) outline a framework created to conceptualize the aging and long-term health implications of vision loss, and (3) discuss the global public health challenges to eye care and to maximizing health for older adults with visual impairments.

